# Radiomics side experiments and DAFIT approach in identifying pulmonary hypertension using Cardiac MRI derived radiomics based machine learning models

**DOI:** 10.1038/s41598-021-92155-6

**Published:** 2021-06-16

**Authors:** Sarv Priya, Tanya Aggarwal, Caitlin Ward, Girish Bathla, Mathews Jacob, Alicia Gerke, Eric A. Hoffman, Prashant Nagpal

**Affiliations:** 1 Department of Radiology, University of Iowa Carver College of Medicine, 200 Hawkins Dr, Iowa City, IA 52242 USA; 2 Department of Family Medicine, University of Iowa Carver College of Medicine, Iowa City, Iowa, USA; 3 Department of Biostatistics, University of Iowa College of Public Health, Iowa City, IA USA; 4 Department of Electrical Engineering, University of Iowa College of Engineering, Iowa City, IA USA; 5 Department of Pulmonary Medicine, University of Iowa Carver College of Medicine, Iowa City, , IA USA; 6 Roy J. Carver Department of Biomedical Engineering, University of Iowa College of Engineering, Iowa City, IA USA

**Keywords:** Machine learning, Cardiology

## Abstract

Side experiments are performed on radiomics models to improve their reproducibility. We measure the impact of myocardial masks, radiomic side experiments and data augmentation for information transfer (DAFIT) approach to differentiate patients with and without pulmonary hypertension (PH) using cardiac MRI (CMRI) derived radiomics. Feature extraction was performed from the left ventricle (LV) and right ventricle (RV) myocardial masks using CMRI in 82 patients (42 PH and 40 controls). Various side study experiments were evaluated: Original data without and with intraclass correlation (ICC) feature-filtering and DAFIT approach (without and with ICC feature-filtering). Multiple machine learning and feature selection strategies were evaluated. Primary analysis included all PH patients with subgroup analysis including PH patients with preserved LVEF (≥ 50%). For both primary and subgroup analysis, DAFIT approach without feature-filtering was the highest performer (AUC 0.957–0.958). ICC approaches showed poor performance compared to DAFIT approach. The performance of combined LV and RV masks was superior to individual masks alone. There was variation in top performing models across all approaches (AUC 0.862–0.958). DAFIT approach with features from combined LV and RV masks provide superior performance with poor performance of feature filtering approaches. Model performance varies based upon the feature selection and model combination.

## Introduction

Radiomics is an emerging subject area that is being increasingly utilized in medical imaging for problems related to classification, prediction and prognosis of various diseases and cancers. The most notable use and development have been made in the field of neurooncology^1–4^. Application of radiomics has recently gained interest in cardiac MRI (CMRI) and few studies have evaluated its utility in patients with ventricular hypertrophy^5–7^, myocardial infarction^8^, tachyarrhythmia^9^, and myocarditis^10,11^.

One of the limitations of radiomics based models is lack of reproducibility and generalizability due to which they have not yet translated to clinical practice. Prior studies show that many radiomic features are unstable and are susceptible to variations during image acquisition, reconstruction, and post processing^12–14^. To improve repeatability and reproducibility, many so-called radiomics side experiments are performed. These side studies or robustness studies are done to identify stable features [assessed by intraclass correlation (ICC) or concordance correlation (CCC)] that are incorporated subsequently in the model creation process after the exclusion of non-stable features. Different strategies for side experiments or stability tests involve scan-rescan, annotation-reannotation of the segmentation masks or doing image perturbations in a small patient subgroup or in a phantom study^15,16^.

However, such feature filtering approach of completely disregarding the unstable features may not be ideal. The mere reliance on identifying stable or reproducible radiomic features helps remove noise from the data, but this may come at the expense of losing relevant information from the original data. As shown by the recent experiments by Gotz et al.^17^, the features that are stable may not be predictive, while the unstable or noisy features may include relevant information essential for prediction. Gotz et al.^17^ proposed a novel approach of data augmentation for information transfer (DAFIT), where instead of filtering unstable features, the results of the side experiments were incorporated into the main data and augmented data sets were created and used for prediction. They showed that the proposed approach when combined with feature selection methodologies led to overall improvement in the model performance compared to simply disregarding the unstable data.

In this study, we build upon the previous work of Gotz et al.^17^ and apply it to the CMRI images of patients with and without pulmonary hypertension (PH). We aim to study the influence of CMRI derived radiomic features from multiple myocardial masks (left ventricle, right ventricle and combined left and right ventricle masks), as well as the impact of multiple side experiments in differentiating patients with and without PH. We compare the predictive performance of the Original data (no inclusion of side study data), radiomic side studies including ICC-based feature filtering, and the DAFIT approach to classify patients with and without PH.

## Results

### Patient demographics

Subjects in the PH group were seen to have higher age, body surface area (BSA), body mass index (BMI), and were more likely to be smokers and have associated diabetes mellitus and hypertension. There were significant differences between the two groups on all variables except sex. Supplementary Tables 1 and 2 present the demographics characteristics available for both groups.

### ICC results

For first two extractions (ICC2), there were 22 features from the LV mask and 46 features from the RV mask that showed excellent ICC. There were only 8 features from LV mask and 24 from RV mask that showed excellent ICC across all three extractions (details in supplementary Table 3).

### Model performance from original data without side studies

The models fit using features from the LV mask had the highest diagnostic performance (AUC 0.921). Table 1 provides the list of top three models from the original data. No significant difference was seen between the performance of the LV and combined (AUC 0.913) masks (p = 0.4702). Significant differences were found between the LV and RV (AUC 0.832) masks (p = 0.0006) and between the RV and combined masks (p = 0.0005, supplementary Table 4). For the PH subgroup, no significant difference (p value > 0.05) was seen between the top performing models (Table 2, supplementary Table 5).

**Table 1:** Top three models selected to fit for entire group (All PH patients versus controls).

Slices	Model	Feature selection	Mean	SD	Median	Min	Max
**Original**							
LV mask	rf	Full	0.921	0.064	0.922	0.750	1.000
LV mask	rf	Corr	0.916	0.069	0.938	0.792	1.000
Combined	rf	Full	0.913	0.054	0.922	0.806	1.000
**First side study—intraclass correlation with first two extractions (ICC2)**							
Combined mask	nnet	Corr	0.905	0.059	0.906	0.750	0.984
Combined mask	nnet	Full	0.904	0.079	0.917	0.708	1.000
Combined mask	mlp	Full	0.904	0.063	0.906	0.750	1.000
**Second side study—intraclass correlation with all three extractions (ICC3)**							
Combined mask	ridge	Full	0.895	0.065	0.891	0.734	0.984
Combined mask	nnet	Full	0.885	0.079	0.903	0.688	1.000
Combined mask	enet	Full	0.873	0.074	0.875	0.734	0.984
**DAFIT without filtering**							
Combined mask	svmPoly	pca	0.958	0.033	0.960	0.846	1.000
Combined mask	svmPoly	Full	0.957	0.043	0.967	0.860	1.000
Combined mask	svmRad	Full	0.939	0.034	0.949	0.853	0.988
**DAFIT with filtering ICC2 (DAFIT Filt2)**							
Combined mask	svmRad	Corr	0.945	0.047	0.945	0.824	1.000
Combined mask	svmPoly	Full	0.943	0.051	0.956	0.816	1.000
Combined mask	svmRad	Full	0.941	0.053	0.945	0.783	1.000
**DAFIT with filtering ICC 3 (DAFIT Filt3)**							
Combined mask	svmRad	Full	0.920	0.060	0.934	0.702	0.978
Combined mask	nnet	Corr	0.903	0.073	0.919	0.625	0.985
Combined mask	svmRad	Corr	0.902	0.075	0.926	0.607	0.989

*LV* left ventricle, *RV* right ventricle, *combined* combined RV and LV masks, *original* original data without inclusion of side experiments, *ICC2* features with excellent intraclass correlation from first two extractions, *ICC3* features with excellent intraclass correlation from all three extractions, *DAFIT* synthetic data creation using main and side study data, *DAFIT Filt2* combining DAFIT with feature filtering from ICC2, *DAFIT Filt3* combining DAFIT with feature filtering from ICC3, *rf* random forest, *nnet* neural network, *mlp* multilayer perceptron, *enet* elastic net, *svmPoly* support vector machine (SVM) with a polynomial kernel, *svmRad* SVM with a radial kernel, *full* full feature set, *corr* high correlation filter, *pca* principal component analysis.

**Table 2:** Top three models selected to fit for PH sub-group (PH subjects with preserved ejection fraction versus controls).

Slices	Model	Feature selection	Mean	SD	Median	Min	Max
**Original**							
RV mask	nnet	Lincomb	0.885	0.097	0.906	0.688	1.000
Combined	rf	Full	0.878	0.092	0.906	0.609	1.000
RV mask	ridge	Lincomb	0.876	0.107	0.906	0.531	1.000
**First side study—interclass correlation with first two extractions (ICC2)**							
Combined mask	gbrm	Full	0.808	0.151	0.844	0.313	1.000
Combined mask	rf	Full	0.798	0.118	0.797	0.594	1.000
RV mask	rf	Full	0.794	0.140	0.813	0.375	1.000
**Second side study—interclass correlation with all three extractions (ICC3)**							
Combined mask	nnet	Full	0.815	0.119	0.813	0.563	1.000
Combined mask	mlp	Full	0.800	0.144	0.844	0.500	1.000
Combined mask	lasso	Full	0.785	0.161	0.750	0.500	1.000
**DAFIT without filtering**							
Combined mask	svmPoly	Full	0.957	0.039	0.969	0.859	1.000
Combined mask	svmPoly	pca	0.947	0.036	0.945	0.891	1.000
Combined mask	svmRad	Full	0.926	0.043	0.930	0.836	0.984
**DAFIT with filtering ICC2 (DAFIT Filt2)**							
Combined mask	svmPoly	Corr	0.908	0.095	0.930	0.617	1.000
Combined mask	svmPoly	Full	0.903	0.098	0.914	0.609	1.000
Combined mask	svmRad	Full	0.890	0.088	0.906	0.617	1.000
**DAFIT with filtering ICC 3 (DAFIT Filt3)**							
Combined mask	svmRad	Full	0.887	0.100	0.906	0.656	0.992
Combined mask	svmRad	Corr	0.881	0.089	0.906	0.648	1.000
Combined mask	linear	Corr	0.863	0.082	0.875	0.703	0.992

*LV* left ventricle, *RV *right ventricle, *combined* combined RV and LV masks, *original* original data without inclusion of side experiments, *ICC2* features with excellent intraclass correlation from first two extractions, *ICC3* features with excellent intraclass correlation from all three extractions, *DAFIT* synthetic data creation using main and side study data, *DAFIT Filt2* combining DAFIT with feature filtering from ICC2, *DAFIT Filt3* combining DAFIT with feature filtering from ICC3, *rf* random forest, *nnet* neural network, *gbrm* gradient boost regression model, *mlp* multilayer perceptron, *enet* elastic net, *lasso* least absolute shrinkage and selection operator, *svmPoly* support vector machine (SVM) with a polynomial kernel, *svmRad* SVM with a radial kernel, *full* full feature set, *corr* high correlation filter, *pca* principal component analysis, *lincomb* linear combinations filter.

### Model performance using features with excellent ICC from first two extractions

The best performing model was built using combined masks (AUC 0.905) and did not differ significantly compared to the best performing combined mask model from the original study (p > 0.05). However, significant differences were seen between the LV (AUC 0.821) and RV (AUC 0.810) mask models built using features from excellent ICC and those built from original data (for LV mask p-value < 0.0001, RV mask p value 0.0382) (Table 1, supplementary Table 6).

For the PH subgroup, a statistically significant decrease in performance was seen for individual and combined masks when using features with excellent ICC compared to the original data (p < 0.05) (Table 2, supplementary Table 7).

### Model performance using features with excellent ICC from all three extractions

This analysis only used features that had excellent ICC between all pairwise combinations of the three feature extractions. Models fit using the combined mask had the best performance (AUC 0.895). No significant performance difference was seen between best model built from the combined and RV (AUC = 0.784) masks using features from ICC3 and original data (p value > 0.05). However, a significant difference was seen between performance of LV mask model built using features from ICC3 and the original data (p-value 0.0012) (Table 1, supplementary Table 8).

For the PH subgroup, a statistically significant decrease in model performance was seen for individual and combined masks when using features from ICC3 compared to the original data (p value < 0.05) (Table 2, supplementary Table 9).

### Model performance from DAFIT approach without feature filtering

The best overall model was built using the combined mask (AUC 0.958) (Table 1, supplementary Table 10). The best model built using the combined masks had statistically significantly better performance compared to models built using individual RV (AUC 0.899) or LV (AUC 0.889) masks (p value < 0.0001). There was not a significant difference in predictive performance between the top models built from individual RV and LV masks (p value = 0.4576).

Similarly, for the PH subgroup, the best model built from the combined masks had significantly higher performance compared to the models built using LV and RV masks (p < 0.05) (Table 2, supplementary Table 11).

### Model performance from DAFIT approach with feature filtering

These models were built only using the combined mask since combined mask was the best overall performer in the previous approaches. The best overall model for combined DAFIT approach and ICC2 achieved an AUC of 0.945. Similarly, for DAFIT approach combined with ICC3, the best model had an AUC of 0.860 (Table 1, supplementary Table 12).

For the PH subgroup analysis, combined DAFIT and feature filtering from ICC2 achieved a mean AUC of 0.908 and for DAFIT and feature filtering from ICC3 the best model had an AUC of 0.887 (Table 2, supplementary Table 13).

### Comparison of model performance across all approaches

**Figure 1:**
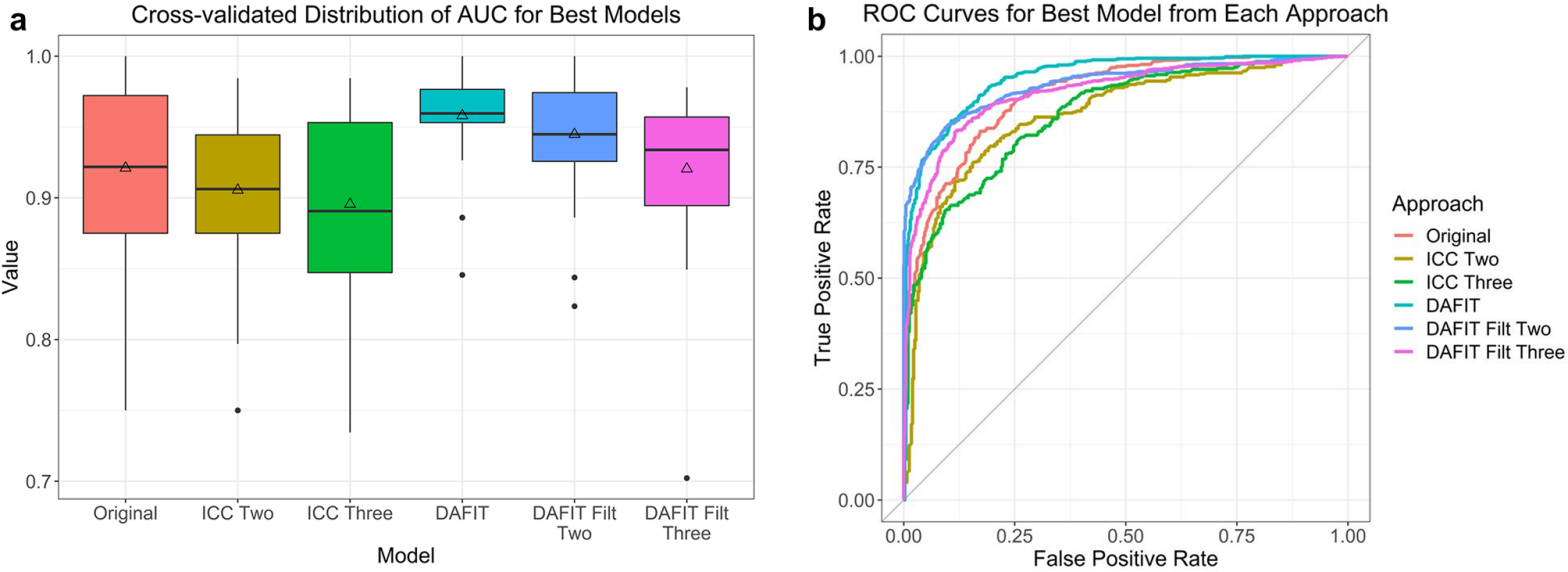
Model predictive performance for primary analysis (all patients with PH and controls). (**a)** The box and whisker plot and **(b)** the ROC curve for multiple approaches analyzed for the primary analysis. DAFIT approach without filtering shows least variation in standard deviation **(a)** and highest area under the curve **(b)**.

DAFIT approach without feature filtering had the highest performance (AUC 0.958) followed by DAFIT with feature filtering (AUC 0.920–0.945). The performance of DAFIT with feature filtering was also higher than original data and ICC2 and ICC3 approaches (Fig. 1a,b).

**Figure 2:**
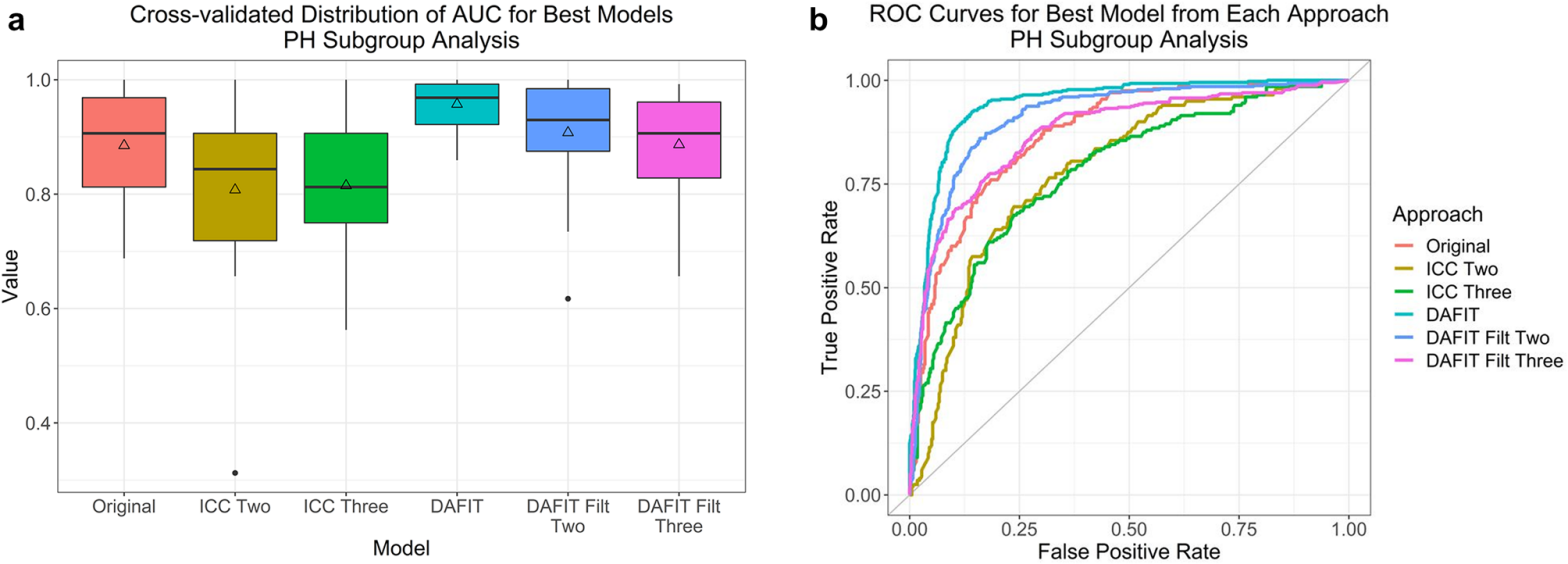
Model predictive performance for subgroup analysis (PH patients with preserved ejection fraction and controls). **(a)** The box and whisker plot and **(b)** the ROC curve for multiple approaches analyzed for the PH subgroup analysis. DAFIT approach without filtering shows least variation in standard deviation **(a)** and highest area under the curve **(b)**.

**Figure 3:**
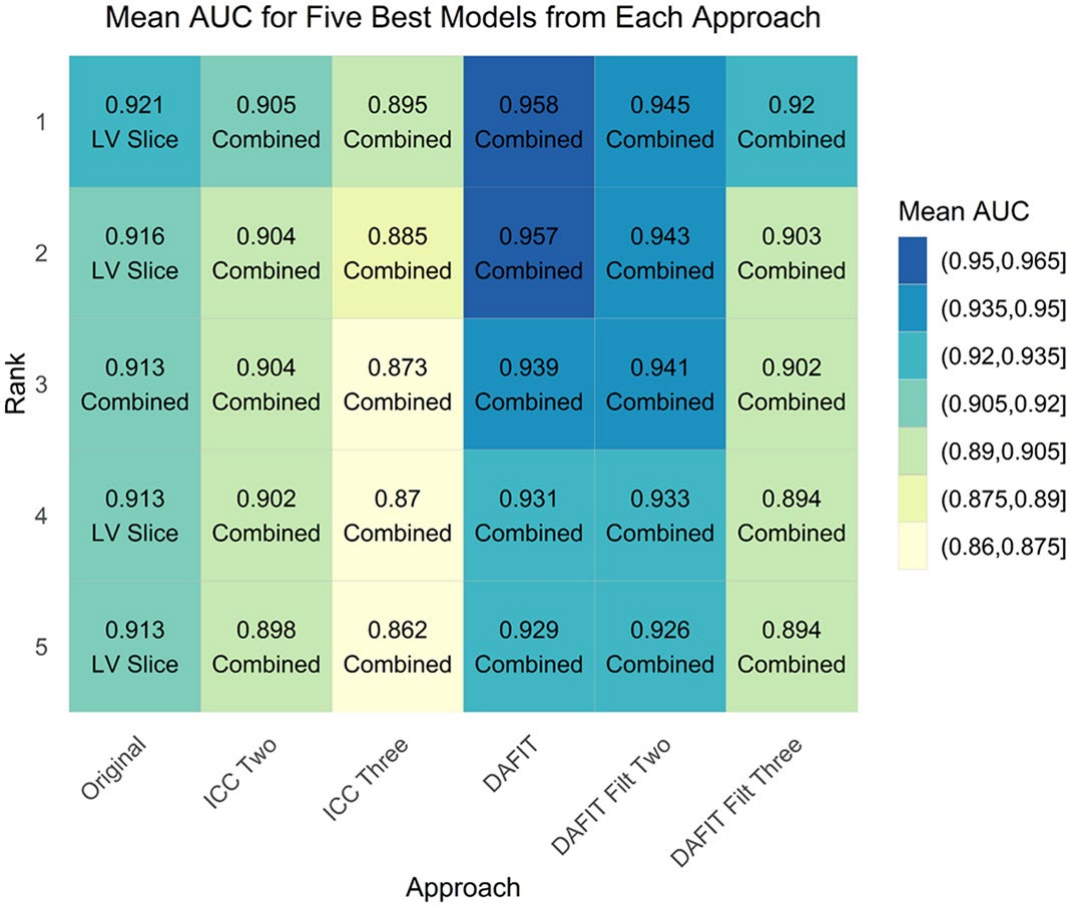
Area under curve for primary analysis (all patients with PH and controls). This figure shows the mean area under the curve for multiple feature selection and model combinations using all approaches for the primary analysis.

**Figure 4:**
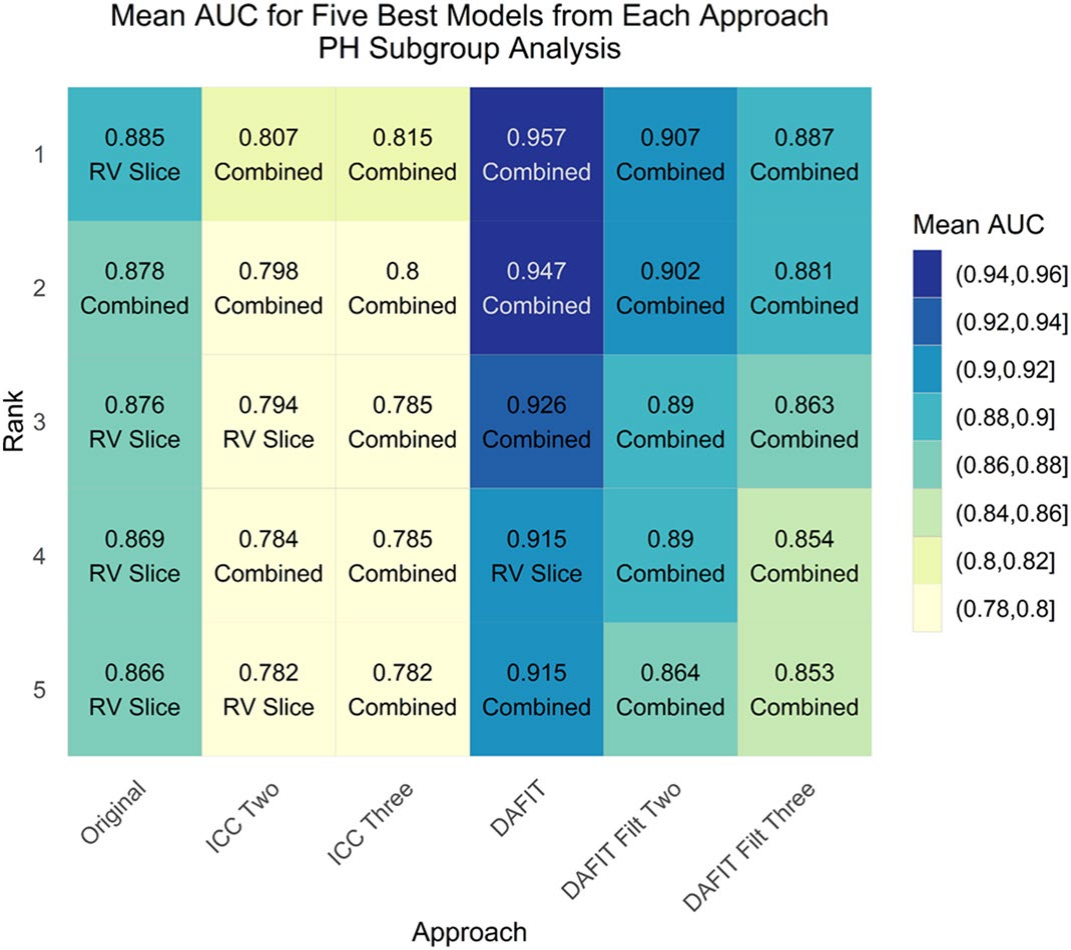
Area under curve for PH subgroup analysis (PH patients with preserved ejection fraction and controls). This figure shows mean area under the curve for multiple feature selection and model combinations using all approaches for the PH subgroup analysis.

For the PH subgroup, DAFIT approach without filtering was again the top performer (AUC 0.957) followed by DAFIT with feature filtering (AUC 0.887–0.907) (Fig. 2a,b). Figures 3 and 4 display the mean AUC for five best models from each approach for primary and PH subgroups respectively.

For the permutation tests assessing model significance, the p-values for the best performing model on all six analysis approaches for both the full and subgroup analyses were 0.0099. This provides strong evidence that the best classifiers across all approaches are able to identify a dependency structure in the data to make accurate predictions of PH subjects.

### Effect of confounder variables

**Figure 5:**
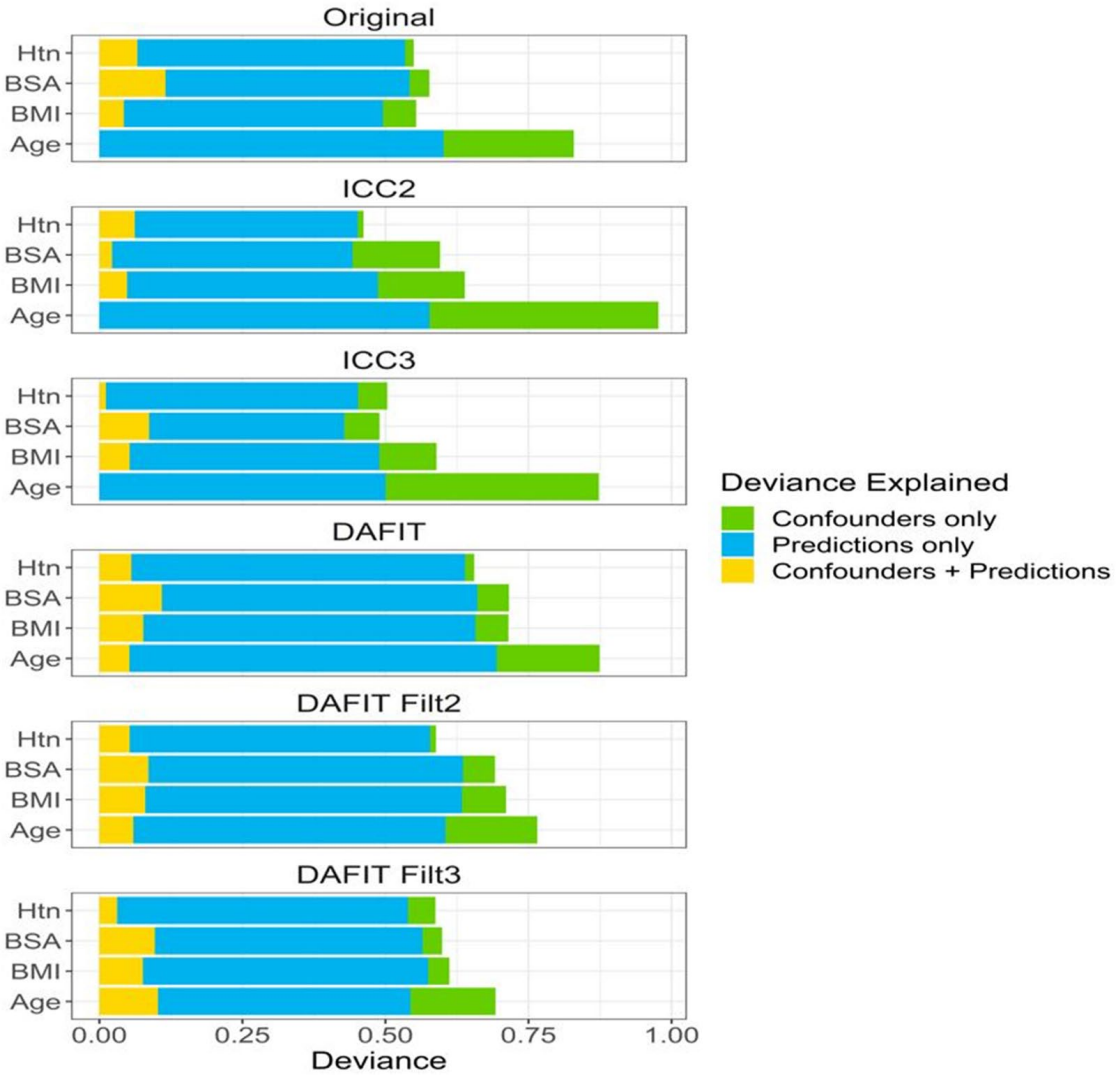
Effect of confounding variables. This figure shows that of all the confounders evaluated, largest amount of deviance in the classifications is explained by age. Also, predictions by machine learning explain a large portion of the deviance that is not explained by the confounder variables. *Htn* hypertension, *BMI* body mass index, *BSA* body surface area, DAFIT—synthetic data creation using main and side study data; DAFIT Filt2—combining DAFIT with feature filtering from ICC2; DAFIT Filt3—combining DAFIT with feature filtering from ICC3.

Of the confounders evaluated (age, BMI, BSA, and presence of hypertension), we see the largest amount of deviance in the classifications is explained by age (Fig. 5). Across all model approaches, the machine learning predictions explain a large portion of the deviance not already explained by the confounder variables. This indicates the high predictive abilities of these models is not fully driven by the confounders. In assessing the statistical significance of adding the model predictions to the confounders, all model/confounder combinations yielded p-values of 0.0099, which is the minimum possible p-value based on 100 permutations. This indicates that using the radiomics features to predict PH offers a significant improvement in performance beyond what could be achieved only from the confounders.

## Discussion

In the current study we studied the impact of six different approaches on the overall model performance. We evaluated LV and RV CMRI radiomics from the original data (without including any side study experiment) and compared that with multiple side studies and recently proposed novel DAFIT approach with and without feature filtering. Our study showed that the DAFIT approach and features extracted from the combined mask provide the highest model performance. We also demonstrated that the models built using only the stable radiomic features lead to suboptimal model performance. Lastly, model performance varied considerably based upon chosen machine learning model and feature selection combination.

Performance of radiomic side studies is encouraged to identify stable features and to improve generalizability since radiomic features are susceptible to variations^18–21^. In terms of side studies, most of the prior work relates to feature filtering involving selection of robust features by virtue of scan-rescan, repeat segmentations or change of image parameters. However, it is difficult to perform repeat scans due to cost and ethical issues of rescanning. Hence, most of the studies select robust features by manually doing reannotation in a subgroup of patient population and identifying features with excellent inter or intraobserver correlation.

However, there is currently no clarity on how to incorporate this information from the side studies into the modelling process. As has been shown in previous studies, the inclusion of only the stable features led to sub-optimal model performance in our study. This again highlights the fact that selecting stable features does not mean better predictive performance. A PET-CT study on nasopharyngeal carcinoma by Lv et al.^22^ also showed that radiomic features with poor ICC may still perform well in disease differentiation. Lv et al.^22^ concluded that low feature stability does not mean poor predictive performance and thus robustness of radiomics features should not be overemphasized. We also observed that the model performance dipped further when we included features that were stable across all three extractions (original data and two repeat annotation side studies) compared to model performance from first two extractions. This finding is important as it raises an interesting question- How many additional side studies one needs to perform? There is no valid answer currently, since overall performance may vary if we perform one, two or even more stability side studies, as also seen in our study.

We also found poor reproducibility of texture features on repeat segmentation. In a recent CMRI-based radiomics study, only 32–73% texture features were found reproducible^23^. This may be secondary to manual segmentation that has been found to have poor reproducibility of features^24,25^.

In contrast to feature filtering approach, the DAFIT approach incorporates information from the side studies without removing unstable features. DAFIT approach incorporates the variability arising from the feature extraction process in creating the augmented data. This allows for important noisy predictors to be retained by utilizing the side study data to accurately represent the true feature generating process.

Another advantage of DAFIT approach lies in its ability to be combined with multiple feature selection and model combinations. We found SVM classifier with a polynomial kernel combined with PCA feature selection as the best overall model while applying DAFIT. In the original study, Gotz et al.^17^ found that LASSO classifier benefitted most from the DAFIT approach when compared to the ensemble methods of random forest and GBRM. However, they did not evaluate any non-linear classifiers. It is difficult to assess the rationale for SVM models performing well with the DAFIT approach. However, as other non-linear approaches performed well when ICC-based feature filtering was used, we posit that the augmentation procedure may increase the non-linear separation which is well-captured by the polynomial and radial kernels. Further studies are needed to verify this finding. Interestingly, the performance of combined DAFIT and feature filtering was superior to original data and side studies but was inferior to DAFIT without filtering. This again provides evidence that feature filtering approach may lead to loss of relevant features from the model building and thus filtering alone may not be ideal.

Data augmentation is a commonly used approach in machine learning to improve classifier performance by enhancing both the size and quality of the available data. For example, when imaging data is used as a model input, each image may be rotated several times to increase the amount of relevant data used in model fitting. In general, data augmentation techniques are known to reduce overfitting by extracting additional information from the available data^26^. In this case of the DAFIT approach, additional information is about the stability of the radiomics features obtained as a result of the side studies. The data is enhanced not only from the increase in the size of the data, but also from the ability to retain relevant features which may be noisy and thus excluded from the filtering approaches. In addition, in their work Gotz et al.^17^ assessed if the improved performance of DAFIT was due to the inclusion of the additional information or only due to the increased sample count and found that when additional artificial samples were used the DAFIT approach had almost identical results.

In terms of myocardial masks, models built using combined masks had significantly higher performance compared to individual masks. At present, there is no clear consensus regarding extraction of myocardial radiomic features from left or right ventricular myocardium or as a combined approach. Though the sample size of our study was small, these results may guide future radiomic studies in selecting the appropriate region of interest.

To remove the effect of low ejection fraction in patients with PH as a confounding variable, we also performed analysis of a subgroup of PH patients with preserved ejection fraction (LVEF ≥ 50%). Our results showed similar performance for the PH subgroup and provides evidence that radiomics features are consistent and are not influenced by the variations in ventricular ejection fraction. These results are, however, specific to the current data set and should be validated on the future studies. Similar to the primary analysis, DAFIT without feature filtering was again the best approach using svmPoly model and full feature set selection in PH subgroup. The performance again dipped when DAFIT was combined with feature filtering, further reiterating that relevant and important features may be lost during filtering.

Our study also demonstrates that the model performance varies considerably based upon chosen model/feature selection combination. Our study is unique as we evaluated combinations of eleven different machine learning classifiers and four different feature selection strategies and found variations even in the top five performing models across all approaches (AUC 0.862–0.958). Prior CMRI radiomic studies have only assessed single or limited machine learning models^10,11,27–30^. We conclude that further studies should also assess multiple models and feature selection strategies to ensure a more rigorous model selection process.

Besides the limitations of retrospective data and small sample size, our study lacks external validation to improve generalizability of the optimal model. However, we performed five-fold cross-validation to avoid bias, minimize overfitting and validate our models. In addition, we performed permutation analysis to test for model significance and found that the top ranked classifiers were able to provide accurate predictions of PH subjects. This was consistent for all six approaches. We extracted radiomics only from mid-ventricular end-systole cine frame. End-systole was selected because it allowed clear depiction of the thin-walled right ventricle. This allowed us to segment RV wall and measure radiomic features from the RV. We selected the mid ventricular short axis to segment the most reproducible mid LV myocardium at the level of papillary muscles. The use of basal and apical slices can induce variability in the research studies and other prior radiomics CMR studies have also used mid-ventricle short-axis for texture measures^31^. Since the primary study goal was to investigate role of novel DAFIT methodology on radiomics feature selection, we believe that use of mid ventricular slice for texture measures is not compromising the results of this study. Another important limitation is the heterogeneity among patient population. The controls were not matched to the PH group regarding age and characteristics such as body mass index, diabetes, or hypertension. This was secondary to the retrospective nature of this study and definition of controls as subjects with normal CMRI. However, we performed regression analysis to evaluate for confounders and found that radiomic features result in a significant improvement in predictive performance beyond confounding variables. Still, future prospective studies should aim to control confounders. In addition, since our study only included healthy controls, the results also need to be validated in prospective studies in a cohort of patients who have no PH on RHC. We also included all WHO (World Health Organization) subtypes of PH. Since majority of PH cases in our study had WHO group 2 (PH secondary to left ventricle dysfunction), our results may not be directly applicable to primary pulmonary hypertension and further studies with larger number of patients from different WHO group PH will be needed to explore if there is any association with primary PH. However, our approach was more pragmatic as selective inclusion of only one class of PH patients may have improved model performance but would have also introduced selection bias.

Validation of recently described novel DAFIT approach, assessing the impact of multiple myocardial masks and several robustness experiments are the primary strengths of our study. We also provide framework for future CMRI radiomic studies regarding feature filtering, radiomic side studies, and machine learning/feature selection strategies.

## Conclusion

Recently proposed novel DAFIT (Data augmentation for information transfer) approach leads to significant improvement in the radiomics model performance when compared to the original data (without including information from side studies) and ICC-based feature filtering approaches. Use of different myocardial masks and the type of side experiment performed changes the CMRI based radiomics model performance for differentiation of patients with and without PH. Models built using combined LV and RV masks perform better than individual masks alone. The performance of machine learning models varies significantly based upon the chosen feature selection and model combinations.

## Methods

This was a retrospective study approved by institutional review board (IRB) of University of Iowa Hospitals (IRB ID # 201811836) and requirement of informed consent was waived off by University of Iowa Hospitals’ IRB. The IRB approved the study, and the study was performed in accordance with Declaration of Helsinki and followed relevant guidelines and regulations. Patients were identified using combination of radiology information system and electronic medical records. Inclusion criteria for the PH group was as follows: CMRI and right heart catheterization (RHC) performed within thirty days of either exam, availability of RHC derived mean pulmonary artery pressure (mPAP)and artifact free balanced steady state free precession (bSSFP) 2D short-axis cine cardiac MRI images. PH was defined as mPAP > 20 mm Hg based on the recent criteria^32^. Since this was a retrospective pilot study, healthy patients were included as the control group as also performed in prior studies^33,34^. A control group was retrospectively selected from patients who underwent CMRI as part of a clinical work-up for a family history of cardiovascular disease. All patients in the control group had a normal CMRI, normal biventricular ejection fraction and no delayed myocardial enhancement. Patients with evidence of ischemic cardiomyopathy, infiltrative cardiomyopathy (like sarcoidosis or amyloidosis), coronary artery disease on CMRI, or cardiac interventions including valve replacement or coronary interventions were excluded from the study (both PH and control group). A total of 82 patients, 42 with PH and 40 controls were included in the final study. The primary analysis included all patients in PH and control groups. A subgroup analysis was also performed that included patients from the PH group who had preserved left ventricle ejection fraction ≥ 50%^35^ and were compared against all controls.

### Cardiac MRI (CMRI)

CMRI was performed on a Siemens 1.5 T MRI (Siemens, Erlangen, Germany). Images were anonymized and were analyzed by two readers with more than five-year experience in cardiac imaging using a FDA approved freely available software “Segment” (version 3.0: http://segment.heiberg.se). The desired end-systolic mid-ventricular short axis^31^ image was selected from the bSSFP short axis cine series for further texture analysis, as performed in prior CMRI studies^31^.

### Right heart catheterization

RHC derived mPAP, pulmonary vascular resistance and pulmonary capillary wedge pressure were recorded.

### Image pre-processing

De-identified DICOM images were transferred to texture software MaZda version 4.6. Image normalization was performed by remapping the histogram data so that the pixels lay within mean ± 3 standard deviations to make sure that the features were reflective of only texture and were not affected by image contrast or overall brightness^19^.

### Image segmentation/mask creation

Segmentation was performed on a mid-ventricular end-systolic slice^31^ by the cardiac imagers (SP and PN) in consensus. Left ventricle (LV) and right ventricle (RV) myocardial masks were manually segmented using the pencil tool within MaZda software. The region of interest (mask) only included the myocardium with exclusion of papillary muscles and the blood pool (Fig. 5).

### Texture features extraction

For each mask, 348 features were extracted using the MaZda software. These included histogram (9), co-occurrence matrix (220), run-length matrix (20), gradient (5), autoregressive (5), geometrical (73) and wavelet (16) features. Details about these features are provided elsewhere and beyond the scope of this work^28,36^.

### Feature repeatability

**Figure 6:**
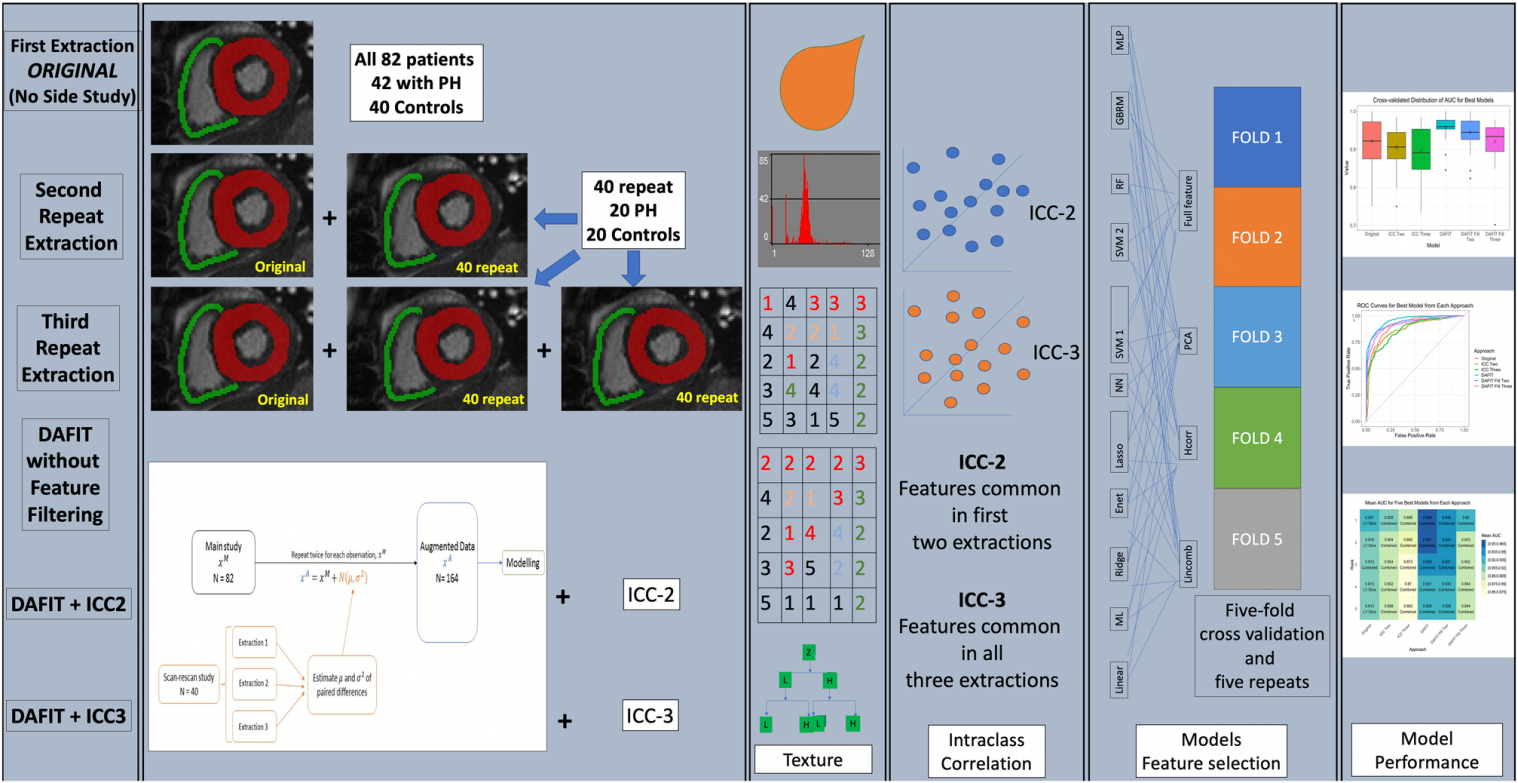
Study workflow. This figure depicts the overall workflow of the study showing radiomics feature extraction from original and multiple side studies, cross-validation and modelling.

To evaluate the repeatability of the texture features, three extractions were performed, with the second and third replications occurring four and six weeks after the original extraction, respectively. This was performed in 40 patients each (20 from each group of PH and control subjects) for repeat extractions (Fig. 6). This was done to evaluate the repeatability of the texture features. Features were recalculated for the 348 radiomic features on both the LV and RV myocardial masks. Intraclass correlation (ICC) was computed to evaluate agreement between the samples for all 348 features from three groups (original mask and two repeated masks) using a two-way mixed-effects model. Based on ICC coefficients, agreement was defined as poor (< 0.40) fair (0.40 to 0.59), good (0.60 to 0.74), and excellent (> 0.75)^37^.

### Radiomics side studies

We investigated six approaches to evaluate different methods of using the side study data: (i) Original—without including the side study experiment data; (ii) ICC2—using only features with excellent ICC based on first two extractions; ((iii) ICC3—using only features with excellent ICC based on all three extractions; (iv) DAFIT—using augmented data generated using all three side study extractions and original data; (v) DAFIT Filt2—using only features with excellent ICC based on two extractions; and (vi) DAFIT Filt3—using only features with excellent ICC based on three extractions (Fig. 6). For approaches (i)–(iv), models were built using features from the individual LV and RV masks as well as combined masks. For approaches (v) and (vi), only combined masks were used.

### Data augmentation for information transfer (DAFIT)

**Figure 7:**
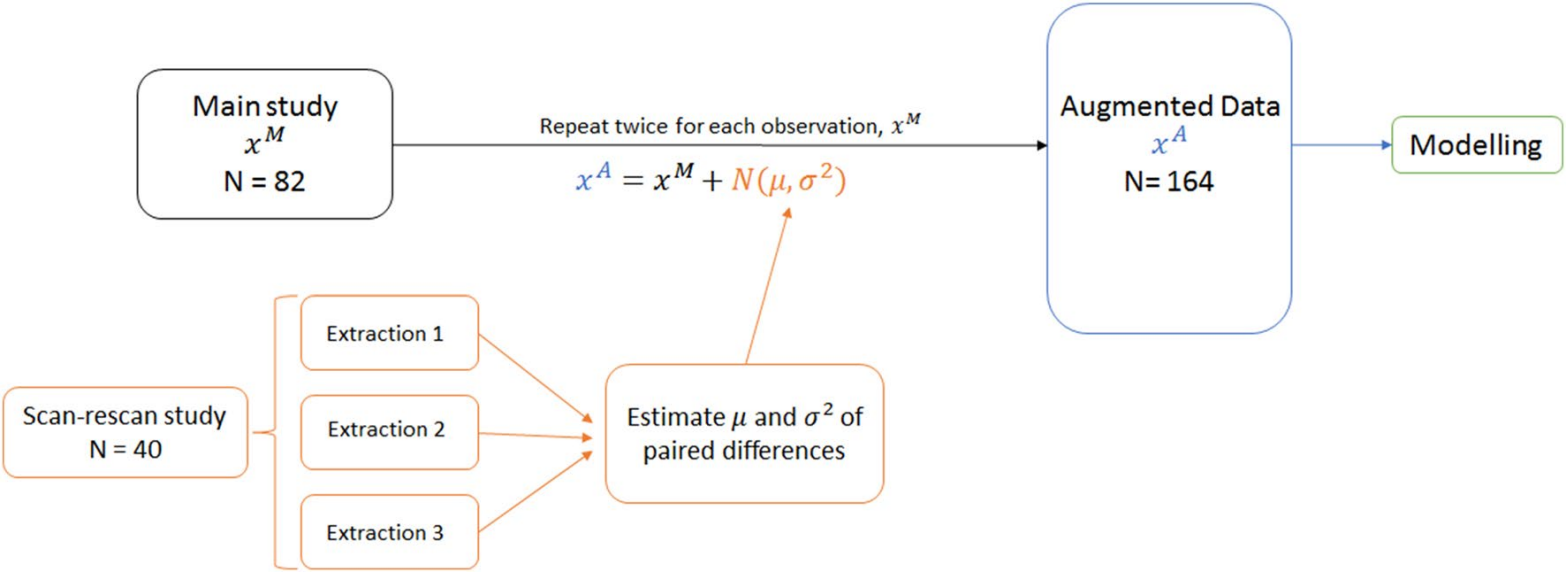
DAFIT (data augmentation with information transfer approach). This figure shows the creation of synthetic augmented data set using DAFIT approach. ${\mathrm{x}}^{\mathrm{M}}$ denotes the original data from the main study, $\mathrm{\mu }$ and ${\mathrm{\sigma }}^{2}$ denote the mean and variance, respectively of the paired differences calculated from the repeated extractions in the side study, and ${\mathrm{x}}^{\mathrm{A}}$ denotes the augmented data.

We utilized the data augmentation procedure as described in the original publication (on DAFIT approach)^17^ for observations from a side study which are paired. We used a transformation of the original data (${x}^{M}$) based on adding random noise generated from a normal distribution with mean and variance defined by the difference between the paired observations. Since we had three feature extractions available from the side studies (three repeat annotated data), three sets of pairwise differences were computed (1–2, 1–3, 2–3) and then the mean and variance ($\mu$ and ${\sigma }^{2}$, respectively) were taken after combining the three sets (Fig. 7). This approach assumes that the differences in the features is solely a result of the extraction process and views the variability as additional noise added to some true value. As was done in the original paper, we generated two augmented observations (${x}^{A}$) from each observation in the main study, yielding 164 observations to be used in the modeling process. The same augmented dataset was used across all models. DAFIT approach was applied both with feature filtering (using results of ICC) and without feature filtering (on original data).

### Feature selection

To reduce dimension for large feature sets for the Original and DAFIT analyses, as well as avoid collinearity across all analyses, three feature selection methods were considered: a linear combinations filter (lincomb), a high correlation filter (corr), and principal components analysis (PCA). The linear combinations filter addresses both collinearity and dimension reduction by finding linear combinations of two or more variables and removing columns to resolve the issue. This process was repeated until the feature set is full rank. As the ICC filtering approaches provide enough a priori feature reduction such that the potential feature set is already full rank, the linear combinations filter was not used in those analyses. The high correlation filter removes variables from the feature set which have a large absolute correlation. A user-specified threshold was given to determine the largest allowable absolute correlation. The number of components retained in the PCA transformation was determined by specifying the fraction of the total variance that should be covered by the components. Table 3 summarizes the thresholds used when considering features from an individual mask or for the combined masks for each of the six approaches. These thresholds were chosen with the goal of sufficiently retaining as much information as possible with enough dimension reduction to allow model fitting. The difference in threshold specifications was due to different sizes of potential feature sets resulting from the six approaches and whether models were fit to a single mask or the combined masks. For all six approaches, we also considered modeling using the entire potential feature set (full) without any specified feature selection techniques. All feature selection methods were implemented using the recipes^38^ package in R version 4.0.2^39^. Prior to any feature selection all variables were standardized.

**Table 3:** Summary of the feature selection and modeling approaches for each analysis (DAFIT Filt analyses were only fit to the combined masks).

	Number of potential features		Lincomb used	Corr thresholds Single mask /combined mask	PCA thresholds Single mask/combined mask
	LV mask	RV mask			
Original	348	348	Yes	0.6/0.5	0.9/0.85
ICC2	22	46	No	0.9/0.9	0.9/0.9
ICC3	8	24	No	–	–
DAFIT	348	348	Yes	0.6/0.4	0.9/0.85
DAFIT Filt2	22	46	No	–/0.8	–/0.85
DAFIT Filt3	8	24	No	–/0.8	–/0.85

*Original* original data without inclusion of side experiments, *ICC2* features with excellent intraclass correlation from first two extractions, *ICC3* features with excellent intraclass correlation from all three extractions, *DAFIT* synthetic data creation using main and side study data, *DAFIT Filt2* combining DAFIT with feature filtering from ICC2, *DAFIT Filt3* combining DAFIT with feature filtering from ICC3, *full* full feature set, *corr* high correlation filter, *pca* principal component analysis, *lincomb* linear combinations filter, *LV *left ventricle, *RV* right ventricle.

### Model fitting and evaluation of predictive performance

Several machine learning algorithms (linear classifiers, non-linear classifiers, and ensemble classifiers) were considered to determine the best classifier for each analysis. The linear classifiers used were linear, logistic, ridge, elastic net, and LASSO regression. The non-linear classifiers used were neural network, support vector machine (SVM) with a polynomial kernel, SVM with a radial kernel, and multi-layer perceptron (MLP). Finally, the ensemble classifiers used were random forest and generalized boosted regression model (GBRM). Models were fit to the full feature set and in combination with feature selection techniques as described in Table 3. In the Original and DAFIT analyses, linear regression, logistic regression, and the neural network were not fit to the full feature set, as it was not full rank, and the neural network was too computationally expensive. In the DAFIT analysis, the linear and logistic regression approaches were also not implemented in combination with the high correlation filter, as even the reduced feature set was not full rank.

### Statistical analysis

Predictive performance of all classifiers was evaluated using fivefold repeated cross-validation with five repeats, resulting in 25 total estimates of performance. For models with tuning parameters, important parameters were adjusted using nested cross-validation to avoid bias. Additionally, the feature selection techniques were carried out within each cross-validated split of the data, so as not to bias the estimate of predictive performance. Model fitting and cross-validated predictive performance was implemented using the MachineShop^40^ and RSNNS^41^ packages in R version 4.0.2^39^. The predictive performance was measured with the area under the receiver operating characteristic curve (AUC) for interpretability. As models were formulated to predict pulmonary hypertension, AUC estimates the probability that a randomly selected subject that had pulmonary hypertension will have a greater predicted value than a randomly selected normal control. Higher AUC values indicate better predictive performance. The model fitting process was also repeated using only the PH subgroup compared to the normal control group. To evaluate the significance of the best performing model from each approach a permutation test was performed using 100 permutations of the data. The permutation test compares the observed measure of predictive performance (AUC) to its null distribution which is obtained by permuting the class labels.

### Effect of confounding variables

To investigate the role of the potential confounding variables age, BMI, BSA, and presence of hypertension, logistic regression models were used to partition the predictive performance of the machine learning models into the components described by the model predictions and the confounder variables^42^. Within each cross-validated test set, logistic regression models were built on the observed classifications using three sets of explanatory variables: confounder variable only, predicted probabilities from the machine learning model only, and confounder and model predictions combined.

On each model, the pseudo R^2^^43^ is computed, providing the fraction of deviance explained by the inclusion of the variables in the model. Decomposing the pseudo R^2^ values, we computed the deviance in the outcome explained by the confounder, the deviance explained by the model predictions, and the deviance explained by the both the confounder and the model predictions. Additionally, we can assess the statistical significance of adding model predictions to what can already by explained by the confounders using the likelihood ratio test (LRT) statistic computed from the models using only the confounder and the confounder and model predictions combined. To account for the lack of independence between cross-validated test sets, the significant of the LRT statistic was evaluated non-parametrically using 100 permutations of the outcome to obtain the null distribution^44^. To combine results across the 25 cross-validated splits, the median deviance explained, and LRT statistic were used. This procedure was repeated for each confounder variable and for the best performing model from each modeling approach for the full analysis. Each confounder was assessed separately to avoid overfitting the logistic regression models.

## Supplementary Information

Supplementary Tables.

## Data Availability

The datasets generated during and/or analyzed during the current study are available from the corresponding author on reasonable request.
