# Supplementary material for: Radiomics side experiments and DAFIT approach in identifying pulmonary hypertension using Cardiac MRI derived radiomics based machine learning models
Source: Sci Rep. 2021 Jun 16;11:12686. doi: 10.1038/s41598-021-92155-6 (PMC8209219; doi:10.1038/s41598-021-92155-6)
Supplement: Supplementary file 1 — Supplementary Tables. [file 41598_2021_92155_MOESM1_ESM.docx]

**Title: Radiomics side experiments and DAFIT approach in identifying pulmonary hypertension using Cardiac MRI derived radiomics based machine learning models**

*Running Title: Radiomics side experiments in Pulmonary Hypertension*

***Authors:***

Sarv Priya^1^, MD, Tanya Aggarwal^2^, MD, Caitlin Ward^3^, MS, Girish Bathla^1^, MD, Mathews Jacob^4^, PhD, Alicia Gerke^5^, MD, Eric A. Hoffman^1,6^, PhD, Prashant Nagpal^1^, MD

^1^Department of Radiology, University of Iowa Carver College of Medicine, Iowa City, Iowa, USA

^2^ Department of Family Medicine, University of Iowa Carver College of Medicine, Iowa City, Iowa, USA

^3^ Department of Biostatistics, University of Iowa College of Public Health, Iowa City, IA, USA.

^4^ Department of Electrical Engineering, University of Iowa College of Engineering, Iowa City, IA, USA.

^5^ Department of Pulmonary Medicine, University of Iowa Carver College of Medicine, Iowa City, Iowa, USA

^6^ Roy J. Carver Department of Biomedical Engineering, University of Iowa College of Engineering, Iowa City, Iowa, USA

**Conflict of Interest:** None

**Funding:** University of Iowa-Carver College of Medicine Small Grant Program (Grant/program #: 53380630; Fund: 243)

**Author Contributions:** SP, TA, GB and PN were involved in project planning, data curation, imaging and radiomics analysis and writing the first draft of the paper. CW performed statistical analysis. MJ, AG and EAH performed provided clinical and advanced imaging expertise and revised the paper. All authors are responsible for revisions and finalization of the paper.

**Corresponding author:**

*Sarv Priya, MD*

Department of Radiology

University of Iowa Carver College Of Medicine,

200 Hawkins Dr, Iowa city, IA, USA 52242

E-mail: [sarv-priya@uiowa.edu](mailto:sarv-priya@uiowa.edu)

Phone: 319-512-5404

**Supplementary Table 1: Demographics, Co-morbidities, and Cardiac MRI features of control and pulmonary hypertension groups**

|  | **Normal**  **(n = 40)** | **Pulmonary Hypertension (PH)**  **(n = 42)** | ***P* value** |
| --- | --- | --- | --- |
| **Age^*^(years)** | 33.5 ± 18.2 | 54.4 ± 17.4 | <0.0001 |
| **Number of Women (%)** | 29 (72.5) | 23 (54.8) | 0.0955 |
| **BMI^*^(** **kg/m2)** | 27.1 ± 7.0 | 34.6 ± 9.0 | <0.0001 |
| **BSA^*^(** **m2)** | 1.8 ± 0.4 | 2.1 ± 0.3 | <0.0001 |
| **RVEF^†^(%)** | 55.0 (52.7-58.2) | 39.5 (29.0-47.7) | <0.0001 |
| **LVEF^†^(%)** | 62.0 (58.0-66.0) | 45.5 (21.0-57.8) | <0.0001 |
| **RVEDVI^†^(ml/m2)** | 75.9 (69.9-86.3) | 97.8 (74-120.7) | 0.0007 |
| **LVEDVI^†^(ml/m2)** | 79.8 (71.4-88.6) | 95.2 (67.4-144.7) | 0.0756 |
| **Smoking Status – n (%)** |  |  | 0.0002 |
| **Current** | 0 (0) | 3 (7.1) |  |
| **Former** | 4 (10) | 19 (45.2) |  |
| **Never** | 36 (90) | 20 (47.6) |  |
| **DM – n (%)** |  |  | 0.0058 |
| **No** | 37 (92.5) | 26 (61.9) |  |
| **Yes** | 3 (7.5) | 15 (38.1) |  |
| **Number with Hypertension (%)** | 10 (25) | 25 (59.5) | 0.0016 |

*BMI: Body mass index; BSA: Body surface area; RVEF: right ventricle ejection fraction; LVEF: Left ventricle ejection fraction; RVEDVI: Right ventricle end-diastolic volume indexed; LVEDVI: Left ventricle end-diastolic volume indexed; DM: Diabetes Mellitus*

**Supplementary Table 2: Right Heart Catheterization (RHC) characteristics of pulmonary hypertension (PH) group with NYHA classification and World Health Organization PH class distribution**

| **Parameters** | **Pulmonary Hypertension (PH)**  **(n = 42)** |
| --- | --- |
| PA Pressure^a^ (mmHg) | 37.0 (22-60) |
| PVR^a^ (Wood Units) | 2.2 (0.9-9.9) |
| PCW^a^ (mmHg) | 22.0 (9-35) |
| Dur b/n RHC and Cardiac MRI (days) | 6.0 (0-30) |
| WHO Class – n (%) |  |
| 1 | 3 (7) |
| 2 | 26 (62) |
| 3 | 1 (2.4) |
| 1 & 2 | 1 (2.4) |
| 1, 2 & 3 | 1 (2.4) |
| 2 & 3 | 9 (21.4) |
| 5 | 1 (2.4) |
| NYHA Class – n (%) |  |
| 1 | 2 (4.7) |
| 2 | 5 (11.9) |
| 3 | 23 (54.7) |
| 4 | 6 (14.3) |
| No | 2 (4.7) |
| Not Available | 4 (9.5) |

^a^ medians w range (min-max) in ()

*PA: Pulmonary artery; PVR: Pulmonary vascular resistance; PCW: Pulmonary capillary wedge pressure; RHC: Right heart catheterization; NYHA: New York Heart Association Classification; WHO:* World Health Organization

**Supplementary Table 3: Intraclass correlation results for two side-studies**

| **ICC Category** | **LV mask** | **RV mask** |
| --- | --- | --- |
| ***First side study*** | | |
| Poor (<0.40) | 215 | 175 |
| Fair (0.40-0.59) | 85 | 63 |
| Good (0.60-0.74) | 26 | 64 |
| Excellent (>0.75) | 22 | 46 |
| ***Second side study (1^st^ vs 3^rd^)*** | | |
| Poor (<0.40) | 297 | 178 |
| Fair (0.40-0.59) | 19 | 69 |
| Good (0.60-0.74) | 19 | 66 |
| Excellent (>0.75) | 13 | 35 |
| ***Second side study (2^nd^ vs 3^rd^)*** | | |
| Poor (<0.40) | 272 | 29 |
| Fair (0.40-0.59) | 46 | 59 |
| Good (0.60-0.74) | 19 | 117 |
| Excellent (>0.75) | 10 | 142 |
| ***Excellent features across all three extractions*** | | |
| Excellent (>0.75) | 8 | 24 |

*LV: Left ventricle; RV: Right ventricle*

**Supplementary Table 4: Performance metrics for best models from original data**

| **Model** | **Feature Selection** | **Mean** | **SD** | **Median** | **Min** | **Max** |
| --- | --- | --- | --- | --- | --- | --- |
| **LV Mask** | | | | | | |
| rf | full | 0.921 | 0.064 | 0.922 | 0.750 | 1.000 |
| rf | corr | 0.916 | 0.069 | 0.938 | 0.792 | 1.000 |
| ridge | full | 0.913 | 0.075 | 0.953 | 0.778 | 1.000 |
| enet | full | 0.913 | 0.072 | 0.917 | 0.750 | 1.000 |
| ada | full | 0.909 | 0.061 | 0.906 | 0.781 | 1.000 |
| **RV Mask** | | | | | | |
| ridge | lincomb | 0.832 | 0.084 | 0.859 | 0.625 | 0.969 |
| svmRad | lincomb | 0.824 | 0.090 | 0.833 | 0.547 | 1.000 |
| lasso | lincomb | 0.822 | 0.087 | 0.828 | 0.594 | 1.000 |
| enet | lincomb | 0.817 | 0.083 | 0.828 | 0.625 | 0.938 |
| svmRad | pca | 0.811 | 0.101 | 0.813 | 0.578 | 0.984 |
| **Combined Mask** | | | | | | |
| rf | full | 0.913 | 0.054 | 0.922 | 0.806 | 1.000 |
| ada | lincomb | 0.899 | 0.076 | 0.922 | 0.736 | 1.000 |
| gbrm | lincomb | 0.891 | 0.080 | 0.906 | 0.694 | 1.000 |
| svmPoly | lincomb | 0.889 | 0.093 | 0.875 | 0.597 | 1.000 |
| svmRad | lincomb | 0.886 | 0.077 | 0.906 | 0.736 | 1.000 |

*Rf: random forest; enet: elastic net; ridge: ridge regression; ada: adaBoost; svmRAD: support vector machine with a radial kernel; lasso: least absolute shrinkage and selection operator; gbrm: generalized boosted regression model; svmPoly: support vector machine with a polynomial kernel; full: full feature set; corr: high correlation filter; lincomb: linear combinations filter; pca: principal component analysis*

**Supplementary Table 5: Performance metrics for best models from original data on pulmonary hypertension subgroup**

| **Model** | **Feature Selection** | **Mean** | **SD** | **Median** | **Min** | **Max** |
| --- | --- | --- | --- | --- | --- | --- |
| **LV Mask** | | | | | | |
| rf | corr | 0.866 | 0.125 | 0.875 | 0.594 | 1.000 |
| mlp | full | 0.865 | 0.116 | 0.906 | 0.594 | 1.000 |
| ridge | full | 0.864 | 0.110 | 0.875 | 0.625 | 1.000 |
| ridge | pca | 0.860 | 0.113 | 0.875 | 0.594 | 1.000 |
| lasso | pca | 0.859 | 0.118 | 0.875 | 0.594 | 1.000 |
| **RV Mask** | | | | | | |
| nnet | lincomb | 0.885 | 0.097 | 0.906 | 0.688 | 1.000 |
| ridge | lincomb | 0.876 | 0.107 | 0.906 | 0.531 | 1.000 |
| enet | lincomb | 0.869 | 0.110 | 0.906 | 0.531 | 1.000 |
| rf | full | 0.866 | 0.083 | 0.875 | 0.719 | 1.000 |
| lasso | lincomb | 0.857 | 0.102 | 0.875 | 0.594 | 0.969 |
| **Combined Mask** | | | | | | |
| rf | full | 0.878 | 0.092 | 0.906 | 0.609 | 1.000 |
| svmRad | pca | 0.858 | 0.096 | 0.875 | 0.656 | 1.000 |
| mlp | full | 0.858 | 0.086 | 0.875 | 0.594 | 1.000 |
| ada | lincomb | 0.845 | 0.109 | 0.844 | 0.563 | 1.000 |
| ada | full | 0.844 | 0.087 | 0.875 | 0.563 | 0.938 |

*Rf: random forest; mlp: multilayer perceptron; lasso: least absolute shrinkage and selection operator; nnet: neural network; enet: elastic net; svmRAD: support vector machine with a radial kernel; ada: adaBoost; full: full feature set; corr: high correlation filter; lincomb: linear combinations filter; pca: principal component analysis*

**Supplementary Table 6: Performance metrics for best models from first side-study**

| **Model** | **Feature Selection** | **Mean** | SD | Median | Min | Max |
| --- | --- | --- | --- | --- | --- | --- |
| **LV Mask** | | | | | | |
| gbrm | corr | 0.821 | 0.104 | 0.833 | 0.569 | 1.000 |
| rf | corr | 0.819 | 0.078 | 0.819 | 0.653 | 0.958 |
| rf | pca | 0.818 | 0.085 | 0.844 | 0.611 | 0.969 |
| gbrm | pca | 0.815 | 0.095 | 0.797 | 0.611 | 1.000 |
| rf | full | 0.806 | 0.078 | 0.813 | 0.653 | 0.938 |
| **RV Mask** | | | | | | |
| rf | full | 0.810 | 0.111 | 0.836 | 0.586 | 1.000 |
| lasso | full | 0.804 | 0.107 | 0.792 | 0.625 | 0.984 |
| lasso | corr | 0.803 | 0.121 | 0.806 | 0.556 | 0.984 |
| rf | corr | 0.802 | 0.100 | 0.806 | 0.617 | 1.000 |
| enet | corr | 0.782 | 0.135 | 0.797 | 0.500 | 1.000 |
| **Combined Mask** | | | | | | |
| nnet | corr | 0.905 | 0.059 | 0.906 | 0.750 | 0.984 |
| nnet | full | 0.904 | 0.079 | 0.917 | 0.708 | 1.000 |
| mlp | full | 0.904 | 0.063 | 0.906 | 0.750 | 1.000 |
| gbrm | full | 0.902 | 0.067 | 0.922 | 0.778 | 1.000 |
| ridge | corr | 0.898 | 0.066 | 0.917 | 0.750 | 0.984 |

*gbrm: generalized boosted regression model; rf: random forest; lasso: least absolute shrinkage and selection operator; enet: elastic net; nnet: neural network; mlp: multilayer perceptron; full: full feature set; corr: high correlation filter; pca: principal component analysis*

**Supplementary Table 7: Performance metrics for best models from first side-study for pulmonary hypertension subgroup**

| **Model** | **Feature Selection** | **Mean** | **SD** | **Median** | **Min** | **Max** |
| --- | --- | --- | --- | --- | --- | --- |
| **LV Mask** | | | | | | |
| gbrm | pca | 0.706 | 0.135 | 0.703 | 0.438 | 0.938 |
| gbrm | full | 0.701 | 0.178 | 0.719 | 0.281 | 1.000 |
| gbrm | corr | 0.701 | 0.160 | 0.719 | 0.281 | 1.000 |
| rf | pca | 0.694 | 0.138 | 0.719 | 0.422 | 0.938 |
| mlp | full | 0.688 | 0.157 | 0.719 | 0.406 | 1.000 |
| **RV Mask** | | | | | | |
| rf | full | 0.794 | 0.140 | 0.813 | 0.375 | 1.000 |
| rf | corr | 0.782 | 0.155 | 0.781 | 0.375 | 1.000 |
| gbrm | full | 0.765 | 0.104 | 0.781 | 0.563 | 1.000 |
| gbrm | corr | 0.763 | 0.120 | 0.750 | 0.531 | 1.000 |
| linear | corr | 0.733 | 0.156 | 0.719 | 0.438 | 0.969 |
| **Combined Mask** | | | | | | |
| gbrm | full | 0.808 | 0.151 | 0.844 | 0.313 | 1.000 |
| rf | full | 0.798 | 0.118 | 0.797 | 0.594 | 1.000 |
| nnet | corr | 0.784 | 0.171 | 0.813 | 0.375 | 1.000 |
| enet | corr | 0.778 | 0.126 | 0.750 | 0.531 | 0.969 |
| rf | corr | 0.774 | 0.137 | 0.781 | 0.500 | 1.000 |

*gbrm: generalized boosted regression model; rf: random forest; mlp: multilayer perceptron; nnet: neural network; enet: elastic net; full: full feature set; corr: high correlation filter; pca: principal component analysis*

**Supplementary Table 8: Performance metrics for best models from second side-study**

| **Model** | **Feature Selection** | **Mean** | **SD** | **Median** | **Min** | **Max** |
| --- | --- | --- | --- | --- | --- | --- |
| **LV Mask** | | | | | | |
| logistic | full | 0.842 | 0.088 | 0.847 | 0.639 | 1.000 |
| linear | full | 0.840 | 0.081 | 0.847 | 0.625 | 0.972 |
| lasso | full | 0.816 | 0.098 | 0.844 | 0.609 | 0.958 |
| ridge | full | 0.815 | 0.089 | 0.833 | 0.578 | 0.922 |
| mlp | full | 0.812 | 0.103 | 0.828 | 0.547 | 0.953 |
| **RV Mask** | | | | | | |
| lasso | full | 0.784 | 0.134 | 0.833 | 0.453 | 0.984 |
| enet | full | 0.772 | 0.127 | 0.797 | 0.484 | 0.969 |
| ridge | full | 0.769 | 0.127 | 0.792 | 0.484 | 0.984 |
| svmPoly | full | 0.766 | 0.126 | 0.766 | 0.438 | 0.984 |
| linear | full | 0.761 | 0.145 | 0.781 | 0.453 | 0.969 |
| **Combined Mask** | | | | | | |
| ridge | full | 0.895 | 0.065 | 0.891 | 0.734 | 0.984 |
| nnet | full | 0.885 | 0.079 | 0.903 | 0.688 | 1.000 |
| enet | full | 0.873 | 0.074 | 0.875 | 0.734 | 0.984 |
| mlp | full | 0.870 | 0.065 | 0.875 | 0.734 | 0.953 |
| svmPoly | full | 0.862 | 0.080 | 0.875 | 0.703 | 0.984 |

*lasso: least absolute shrinkage and selection operator; mlp: multilayer perceptron; svmPoly: support vector machine with a polynomial kernel; nnet: neural network; enet: elastic network; full: full feature set*

**Supplementary Table 9: Performance metrics for best models from second side-study for pulmonary hypertension subgroup**

| **Model** | **Feature Selection** | **Mean** | **SD** | **Median** | **Min** | **Max** |
| --- | --- | --- | --- | --- | --- | --- |
| **LV Mask** | | | | | | |
| linear | full | 0.693 | 0.148 | 0.656 | 0.469 | 1.000 |
| logistic | full | 0.689 | 0.152 | 0.688 | 0.500 | 1.000 |
| ridge | full | 0.673 | 0.167 | 0.625 | 0.375 | 1.000 |
| enet | full | 0.655 | 0.163 | 0.625 | 0.406 | 1.000 |
| mlp | full | 0.650 | 0.170 | 0.656 | 0.281 | 0.938 |
| **RV Mask** | | | | | | |
| nnet | full | 0.764 | 0.127 | 0.781 | 0.531 | 1.000 |
| enet | full | 0.738 | 0.116 | 0.750 | 0.531 | 0.969 |
| ada | full | 0.736 | 0.137 | 0.750 | 0.469 | 1.000 |
| ridge | full | 0.736 | 0.143 | 0.781 | 0.469 | 1.000 |
| svmPoly | full | 0.736 | 0.165 | 0.750 | 0.375 | 1.000 |
| **Combined Mask** | | | | | | |
| nnet | full | 0.815 | 0.119 | 0.813 | 0.563 | 1.000 |
| mlp | full | 0.800 | 0.144 | 0.844 | 0.500 | 1.000 |
| lasso | full | 0.785 | 0.161 | 0.750 | 0.500 | 1.000 |
| ridge | full | 0.785 | 0.132 | 0.813 | 0.531 | 1.000 |
| svmPoly | full | 0.783 | 0.189 | 0.781 | 0.281 | 1.000 |

*enet: elastic net; mlp: multilayer perceptron; nnet: neural network; ada: adaBoost; svmPoly: support vector machine with a polynomial kernel; full: full feature set*

**Supplementary Table 10: Performance metrics for best models from DAFIT approach without feature filtering**

| **Model** | **Feature Selection** | **Mean** | SD | Median | Min | Max |
| --- | --- | --- | --- | --- | --- | --- |
| **LV Mask** | | | | | | |
| svmPoly | full | 0.889 | 0.051 | 0.893 | 0.779 | 0.963 |
| rf | full | 0.888 | 0.062 | 0.895 | 0.735 | 0.988 |
| rf | corr | 0.883 | 0.054 | 0.888 | 0.767 | 0.978 |
| ada | full | 0.872 | 0.055 | 0.864 | 0.779 | 0.974 |
| enet | full | 0.867 | 0.051 | 0.857 | 0.772 | 0.974 |
| **RV Mask** | | | | | | |
| svmPoly | pca | 0.899 | 0.057 | 0.901 | 0.783 | 0.992 |
| svmPoly | full | 0.885 | 0.060 | 0.886 | 0.765 | 0.973 |
| svmRad | full | 0.870 | 0.062 | 0.879 | 0.713 | 0.980 |
| svmPoly | corr | 0.865 | 0.075 | 0.871 | 0.676 | 0.973 |
| mlp | full | 0.845 | 0.048 | 0.855 | 0.757 | 0.938 |
| **Combined Mask** | | | | | | |
| svmPoly | pca | 0.958 | 0.033 | 0.960 | 0.846 | 1.000 |
| svmPoly | full | 0.957 | 0.043 | 0.967 | 0.860 | 1.000 |
| svmRad | full | 0.939 | 0.034 | 0.949 | 0.853 | 0.988 |
| svmRad | pca | 0.931 | 0.035 | 0.938 | 0.864 | 1.000 |
| mlp | pca | 0.929 | 0.034 | 0.941 | 0.864 | 1.000 |

*svmPoly: support vector machine with a polynomial kernel; rf: random forest; ada: adaBoost; enet: elastic net; svmRad: support vector machine with a radial kernel; mlp: multilayer perceptron; full: full feature set; corr: high correlation filter; pca: principal component analysis*

**Supplementary Table 11: Performance metrics for best models from DAFIT approach for pulmonary hypertension subgroup without feature filtering**

| **Model** | **Feature Selection** | **Mean** | **SD** | **Median** | **Min** | **Max** |
| --- | --- | --- | --- | --- | --- | --- |
| **LV Mask** | | | | | | |
| lasso | corr | 0.875 | 0.068 | 0.883 | 0.688 | 0.945 |
| lasso | full | 0.857 | 0.060 | 0.875 | 0.734 | 0.953 |
| enet | corr | 0.856 | 0.064 | 0.852 | 0.688 | 0.961 |
| enet | full | 0.854 | 0.069 | 0.875 | 0.688 | 0.945 |
| gbrm | corr | 0.829 | 0.085 | 0.844 | 0.633 | 0.969 |
| **RV Mask** | | | | | | |
| svmPoly | pca | 0.915 | 0.070 | 0.930 | 0.734 | 1.000 |
| svmPoly | full | 0.907 | 0.067 | 0.914 | 0.742 | 1.000 |
| svmPoly | corr | 0.892 | 0.071 | 0.898 | 0.773 | 1.000 |
| svmRad | full | 0.871 | 0.069 | 0.883 | 0.695 | 0.984 |
| svmRad | corr | 0.863 | 0.074 | 0.859 | 0.695 | 0.984 |
| **Combined Mask** | | | | | | |
| svmPoly | full | 0.957 | 0.039 | 0.969 | 0.859 | 1.000 |
| svmPoly | pca | 0.947 | 0.036 | 0.945 | 0.891 | 1.000 |
| svmRad | full | 0.926 | 0.043 | 0.930 | 0.836 | 0.984 |
| ridge | full | 0.915 | 0.048 | 0.914 | 0.797 | 0.984 |
| svmRad | pca | 0.903 | 0.053 | 0.922 | 0.805 | 0.984 |

*lasso: least absolute shrinkage and selection operator; enet: elastic net; gbrm: generalized boosted regression model; svmPoly: support vector machine with a polynomial kernel; svmRad: support vector machine with a radial kernel; full: full feature set; corr: high correlation filter; pca: principal component analysis*

**Supplementary Table 12: Summary statistics for AUC for top five models built using DAFIT and features with feature filtering in all patients**

| **Excellent ICC from first two extractions** | | | | | | |
| --- | --- | --- | --- | --- | --- | --- |
| **Model** | **Feature Selection** | **Mean** | **SD** | **Median** | **Min** | **Max** |
| svmRad | corr | 0.945 | 0.047 | 0.945 | 0.824 | 1.000 |
| svmPoly | full | 0.943 | 0.051 | 0.956 | 0.816 | 1.000 |
| svmRad | full | 0.941 | 0.053 | 0.945 | 0.783 | 1.000 |
| svmPoly | corr | 0.933 | 0.055 | 0.952 | 0.801 | 1.000 |
| svmPoly | pca | 0.926 | 0.070 | 0.941 | 0.717 | 1.000 |
| **Excellent ICC from all three extractions** | | | | | | |
| **Model** | **Feature Selection** | **Mean** | **SD** | **Median** | **Min** | **Max** |
| svmRad | full | 0.920 | 0.060 | 0.934 | 0.702 | 0.978 |
| nnet | corr | 0.903 | 0.073 | 0.919 | 0.625 | 0.985 |
| svmRad | corr | 0.902 | 0.075 | 0.926 | 0.607 | 0.989 |
| nnet | full | 0.894 | 0.071 | 0.890 | 0.647 | 0.971 |
| svmPoly | full | 0.894 | 0.070 | 0.915 | 0.739 | 0.977 |

*svmPoly: support vector machine with a polynomial kernel; svmRad: support vector machine with a radial kernel; nnet: neural network; full: full feature set; corr: high correlation filter; pca: principal component analysis*

**Supplementary Table 13: Summary statistics for AUC for top five models built using DAFIT and features with feature filtering in PH subgroup**

| **Excellent ICC from first two extractions** | | | | | | |
| --- | --- | --- | --- | --- | --- | --- |
| **Model** | **Feature Selection** | **Mean** | **SD** | **Median** | **Min** | **Max** |
| svmPoly | corr | 0.908 | 0.095 | 0.930 | 0.617 | 1.000 |
| svmPoly | full | 0.903 | 0.098 | 0.914 | 0.609 | 1.000 |
| svmRad | full | 0.890 | 0.088 | 0.906 | 0.617 | 1.000 |
| svmRad | corr | 0.890 | 0.068 | 0.898 | 0.758 | 0.977 |
| svmPoly | pca | 0.864 | 0.081 | 0.883 | 0.625 | 0.984 |
| **Excellent ICC from first three extractions** | | | | | | |
| **Model** | **Feature Selection** | **Mean** | **SD** | **Median** | **Min** | **Max** |
| svmRad | full | 0.887 | 0.100 | 0.906 | 0.656 | 0.992 |
| svmRad | corr | 0.881 | 0.089 | 0.906 | 0.648 | 1.000 |
| linear | corr | 0.863 | 0.082 | 0.875 | 0.703 | 0.992 |
| linear | full | 0.854 | 0.089 | 0.852 | 0.664 | 0.992 |
| svmPoly | full | 0.853 | 0.098 | 0.867 | 0.625 | 0.984 |

*svmPoly: support vector machine with a polynomial kernel; svmRad: support vector machine with a radial kernel; full: full feature set; corr: high correlation filter; pca: principal component analysis*
